# Comment on “Effective Range of Percutaneous Posterior Full-Endoscopic Paramedian Cervical Disc Herniation Discectomy and Indications for Patient Selection”

**DOI:** 10.1155/2020/3548194

**Published:** 2020-04-04

**Authors:** Jun-Song Yang, Lei Chu, Hao Chen, Peng Liu, Ding-Jun Hao

**Affiliations:** ^1^Department of Spine Surgery, Honghui Hospital, Xi'an Jiaotong University, No. 76 Nanguo Road, Xi'an, Shaanxi 710054, China; ^2^Department of Orthopedics, The Second Affiliated Hospital, Chongqing Medical University, Chongqing, China

We read with great interest the article by Wen et al. [1], concerning the effective range of percutaneous posterior full-endoscopic paramedian cervical disc herniation discectomy and indications for patient selection.

We would like to congratulate the authors for their interesting paper, but we would like to make some comments because we are a little bit concerned with the measurement method they apply to define the vertical distance between the lateral border of the dural sac and the peak of the herniated disc (DSPHD); the vertical distance between the lateral border of the dural sac and the intersection of the dural sac and the medial border of the herniated disc (DSMHD); and the vertical distance between the lateral border of the dural sac and the intersection of the dural sac and the medial border of discectomy (DSMD).

To the patients with huge paramedian cervical disc herniation, the lateral border of the dural sac usually becomes obscured (Figure 1), which could not be easily and accurately delineated as shown in the figure. The medial margin of the uncovertebral joint seems to be more appropriate (red arrow). DSMD is measured based on the magnetic resonance imaging (MRI) at 3 days after surgery. Different from the traditional open or microendoscopic discectomy, percutaneous endoscopic surgery is performed under the continuous saline irrigation. The evaluation of the region of discectomy is likely to be affected by the residual fluid and the adjacent edematous tissue. In order to avoid the interference from the residual fluid, the axial T1-weighted MRI seems to be more appropriate to locate the medial border of discectomy at the early stage postoperatively.

In the postoperative follow-up, the authors found that the distance between the edge of the dural sac and the inside edge of the intervertebral disc was significantly smaller than between the edge of the dural sac and the inside edge of the herniated disc. It should be that postoperative DSMD is less than the preoperative DSMHD.

They considered the retraction of the protruding nucleus pulposus after the intradiscal decompression and explained why the incomplete removal of the nucleus pulposus also resulted in the improvement of clinical outcome. We believed that the phenomenon should be verified by further MRI measurement. Additionally, we considered that the symptoms' relief was also related with the indirect neural decompression. After foraminal unroofing and resection of ligamentum flavum, the spinal canal was enough to accommodate the endoscope and available for the spinal cord and nerve root to compensate the compression from the ventral-protruded nucleus pulposus.

## Figures and Tables

**Figure 1:**
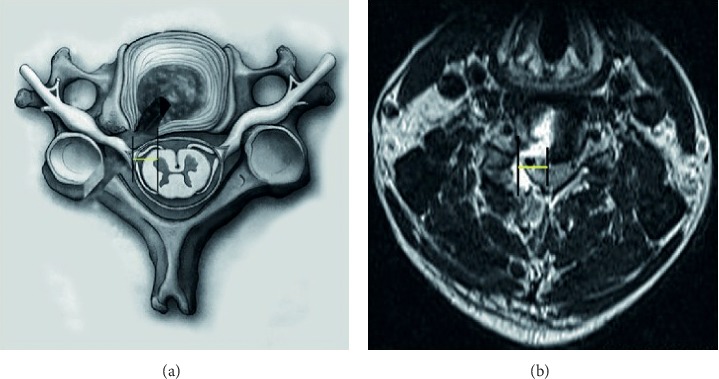
The lateral border of the dural sac and the peak of the herniated disc (DSPHD; red line) and the lateral border of the dural sac and the intersection of the dural sac and the medial border of the herniated disc (DSMHD; blue line) (a) and an MRI image (b). The medial margin of the uncovertebral joint was marked by the red arrow.
